# Changing educational gradient in long-term care-free life expectancy among German men, 1997-2012

**DOI:** 10.1371/journal.pone.0222842

**Published:** 2019-09-19

**Authors:** Olga Grigoriev, Gabriele Doblhammer

**Affiliations:** 1 Max Planck Institute for Demographic Research, Rostock, Germany; 2 Institute for Sociology and Demography, University of Rostock, Rostock, Germany, German Center for Neurodegenerative Disease, Bonn, Germany; Catholic University of Korea College of Medicine, REPUBLIC OF KOREA

## Abstract

**Background:**

The inverse association between mortality and individual socioeconomic status is well-documented. Due to the lack of appropriate data, little is known about the nature of this association among individuals with long-term care (LTC) needs.

**Objectives:**

We aim to fill in this knowledge gap by estimating life expectancy (LE), life expectancy without (CFLE) and with (CLE) long-term care by education for older German men; and by assessing the trends in the education-LE/CFLE/CLE gradient over time.

**Data and methods:**

We apply survival analysis and Gompertz regression to German Socioeconomic Panel data (1997–2012) to estimate the mortality levels and to construct the life tables for three educational categories. Using the administrative data from the health insurance, we adjust mortality rates upward to account for the institutionalized population. We estimate age-specific LTC prevalence from the German Microcensus data (2004, 2012) and compute life expectancy with and without LTC by employing Sullivan’s method. Slope and Relative Indices of Inequality are computed to evaluate the magnitude of educational inequalities in CFLE.

**Results:**

There is a clear and growing educational gradient in LE and CFLE among older men in Germany. In 2004, LE at age 65 among men with low education was 14.2 years, or 3.3 years lower than among highly educated individuals. The CFLE of these two educational categories ranged from 13.6 to almost 17 years. The gradient increased over time and in 2012 the difference constituted 4.6 years. The gaps between educational groups were not pronounced for CLE. The declining health ratio of years without LTC to remaining LE suggests the expansion of LTC needs, irrespective of the educational level.

**Conclusions:**

Growing inequalities by educational status among older German men with care needs demand the attention of policy-makers. Prompt actions are needed to increase the survival chances of the most vulnerable groups.

## Introduction

### Theoretical background

A vast body of literature has investigated the association between mortality and health on the one hand and education on the other [1–11]. Previous research has confirmed that individuals with higher socioeconomic status (SES) have lower mortality risks regardless the SES measure (education, income or occupation) used.

The accumulated evidence demonstrates that the SES-health gradient applies not only to the working-age population, but also to the older individuals. The self-perceived health, mental well-being, and functional limitations of people at higher ages are affected by their education, income, and previous occupation [12–15].

In Germany, efforts to study mortality differentials by SES have been hampered by the lack of information on socioeconomic status on death certificates [5]. Due to strict data protection rules, the linkage of individual records is not permitted in Germany. Such linkages have, however, been performed in many other countries (e.g., for Austria [16–18]; for Switzerland [19–20]; for Finland [21–22]; for Norway [23] and for Finland and Norway [24]; for Lithuania [25–26]; and for Belgium [27]).

Despite these data availability constraints, several existing studies have assessed mortality differentials in Germany. They were based on either sample surveys or data from the German Federal Pension Fund, and used different SES indicators [5, 28–35]. Luy and colleagues [5] used data from the German Life Expectancy Survey to estimate the differences in length of life among West German citizens by education, household income, and occupation. They highlighted the lack of national mortality data by socioeconomic status in Germany, and illustrated that there are alternative ways of estimating life expectancy using survey data with a mortality follow-up. The authors were able to estimate life expectancy at ages 40 and 65, as well as the probability of surviving between both ages separately for each SES dimension. The results revealed substantial differences in life expectancies across all SES groups. For example, the gap in life expectancy between the highest and the lowest educated men aged 65 or older was 3.7 years.

Kroll and Lampert [30] used the German Socioeconomic Panel (GSOEP) data for the estimation of differences in life expectancy, while taking income into account. They used survival models with exponential baseline hazards to estimate relative mortality risks for different socioeconomic groups, and combined them with official life tables for Germany. The individual’s age was introduced into the model as the covariate, in addition to five distribution-based income groups. The mean net equivalent income was used for categorizing income, where the lowest category (less than 60% of the mean equivalent income) represented the group with a relatively high poverty risk, and the highest category (more than 150% of the mean equivalent income) represented the group with a relatively high level of material prosperity. The analysis was done separately for men and women. Their results revealed a clear relationship between income and mortality, with higher income groups having higher life expectancy. The life expectancy of men at age 65 in the highest income group was estimated to be 23.5 years, or 7.9 years higher than that of their counterparts in the lowest income group. Similarly, the life expectancy of women at age 65 with the lowest income was estimated to be 17.9 years, or 6.8 years lower than that of the women in the highest income category. However, the authors acknowledged the general problem that life expectancy based on the GSOEP data may be overestimated.

Doblhammer et al. [35] used the GSOEP data for 1991–2006 to evaluate the effect of family status, education, monthly income, professional position, household size, and health satisfaction on the life expectancy of men and women aged 50 or older. The authors applied the Gompertz model to estimate the relative risks for all covariates, and then the age-specific mortality rates. In addition, they used the estimates for smoking, drinking, blood pressure, and diabetes from the available literature. Their results on the differences in life expectancy by education for both men and women were similar to those of previous studies.

For many European countries, it has been demonstrated that relative inequalities in mortality have increased in recent decades [3,17,36–38]. In general, this increase appears to be more pronounced for men than for women. The findings on the changes in absolute inequalities differ: some studies have reported no change [39], while others have suggested that these inequalities have been narrowing over time [38]. By contrast, Schumacher and Vilpert [10] found that in Switzerland between 1990–1995 and 2000–2005, educational differentials remained almost constant in relative terms, but increased in absolute terms.

While the number of comparative studies for European countries on the trends in SES disparities in overall mortality and from specific causes has been growing [3,36,38,40–41], Germany is not included in any of them. Most of these studies have explored either the social gradient in mortality or in health, but some have combined these two indicators into healthy life expectancy. Previous research has also confirmed the existence of a reverse relationship between education and poor health, as approximated by the ability to perform activities of daily living and self-rated levels of health disability [13,42]. Moreover, a systematic association between education and healthy life expectancy has been confirmed, whereby better educated people can expect to live longer and to have more years in good health than people with less education. It has also been shown that the SES gradient is much steeper for health expectancy than for life expectancy [22,24,43].

While it is difficult to estimate the magnitude of the social gradient in German mortality, it is even more problematic to explore the differences in levels of long-term care need in Germany by educational attainment; again due to the lack of appropriate data. Even the data collected by the census on mandatory LTC insurance [44–45] do not include information on the education of individuals.

Scholz [46] estimated long-term care-free life expectancy at birth and age 60 for German men and women using the official long-term care statistics and data from the Human Mortality Database (HMD) for 2013. The results indicated that, on average, men aged 60 or older can expect to live another 21.38 years, of which 19.43 years will be LTC-free and 1.95 years will be with care needs.

Kreft and Doblhammer [44] reviewed selected publications on the trends in LTC needs in Germany, and highlighted the inconsistency of the findings. They reported that some studies found evidence supporting the compression of LTC, while others found evidence supporting either a dynamic equilibrium scenario or the expansion in LTC in recent decades in Germany. The authors used administrative census data covering all German LTC insurance beneficiaries (2001–2009) to estimate CFLE and CLE by different care need levels (any or severe) for 412 counties. Their results indicate that in the majority of these counties, there has been an expansion of any care needs, but a compression of life years with severe care needs. The authors also demonstrated that mortality, and not morbidity, has been the driving force in the absolute changes in CFLE and CLE.

### Long-term care insurance in Germany

To measure care need, we use information on individuals receiving benefits from the mandatory German long-term care insurance system (LTCI, introduced in Germany in 1995), which also covers people with mandatory private health insurance (detailed descriptions of the LTCI scheme and its changes over time are available elsewhere [47–51]). According to data from the Federal Ministry of Health [47], at the end of 2017 there were 3.302 million beneficiaries of mandatory social long-term care insurance, of whom 77.3% were aged 65 or older. The data further showed that 1.247 million recipients of LTC were men, 66.9% of whom were aged 65 or older (for women, the corresponding figures were 2.055 million recipients and 83.7%). In addition, there were an estimated at 0.189 million private LTCI recipients at the end of 2016.

In December 2015, about three-quarters (73%) of people in need of LTC were cared for at home, while the other 27% were living in a care home [52]. Women represented 61% of those cared for at home and 72% of those living in a home for the older individuals. The oldest old, or those aged 85 or older, represented 32% of those receiving help at home and half of those living in a care home.

The Federal Ministry of Health [47] has projected that the number of LTC benefit recipients will increase to 5.32 million by 2050. It seems likely, however, that since LTCI provides only partial support, whereas additional care expenses must be paid privately [50–51], an individual’s socioeconomic status (SES) will have a large impact on his or her use of care. The results of a previous analysis on the effect of education on the incidence and prevalence of LTC [53] confirmed that educational attainment matters for the utilization of care; i.e., that older adults with higher educational levels have a lower incidence and prevalence of LTC use than their less educated counterparts. The effect was found to be weaker for incidence than for prevalence.

### Objectives of the study

The present work aims to examine the relationship between SES and life expectancy with and without LTC needs among older adults in Germany. It also evaluates the changes in the SES–LE/CFLE/CLE gradient over the 1997–2012 period. As a proxy for socioeconomic status we use education, which is known to be a reliable predictor of health and mortality [54]. Like earlier studies on mortality gradient in Germany [33–34,55], we focus here on estimating the results for men only. One of the reasons why we did not analyze the results for women (provided in S1 Appendix) is the lack of consistency in the mortality estimates from GSOEP for the second period (see the strengths and limitations section for more details).

Based on previous research, we hypothesize that in Germany, the share of life expectancy in which individuals require long-term care in overall life expectancy is rising over time (i.e., that there is an expansion of LTC). We also expect to observe a strong educational gradient in mortality and care needs that tends to increase over time.

## Data and methods

Our analysis applies anonymized data from three main datasets: the German Socioeconomic Panel [56], the German Microcensus (MC [57]), and the administrative data of the largest German public health insurance fund, the “Allgemeine Ortskrankenkassen” (AOK). The GSOEP is approved to be in agreement with the standards of the Federal Republic of Germany for lawful data protection. The GSOEP data are collected by the German Institute for Economic Research (DIW) and provided as scientific use files. The MC data can be ordered at the Research Data Center of the Federal Statistical Office also as scientific use files (or de-facto anonymised 70 percent subsample of the households in the MC). The use of anonymized administrative health claims data from the AOK that never involved patients directly has been approved by the scientific research institute of the AOK (WldO).

The GSOEP is an ongoing survey of individuals, families, and households that covers various demographic and socioeconomic domains (e.g., labor market, education, occupation, health, and life satisfaction). The panel began in 1984, with about 12,000 individuals aged 16 or older living in West Germany, and was extended to cover all of Germany after reunification [58–59]. Respondents remain under observation until they die or drop out. The information about survey-related panel attrition and about the frequency of successful follow-ups is described elsewhere [60].

We use the GSOEP data to estimate the age–specific mortality rates for men aged 65 or older by educational category; to construct life tables; and to obtain life expectancy estimates. The estimates are produced for two periods (1997–2004 and 2005–2012). The GSOEP sample is restricted to individuals who participated in at least two survey years and were aged 65 or older in the two corresponding periods. We observed respondents after their first interview, and censored them at the end of the follow-up period if they had not previously passed away. All observations with incomplete information on censoring or on the dependent variable are excluded. The innovative and high-income sub-samples (F and G) are excluded from the analysis, as they have different selection procedures. To ensure data consistency across the two analyzed periods, the refreshment samples added after 2004 (H, I, J, and K) were excluded. Only those samples that were available in both cross-section datasets are used. The samples consist of 2,052 individuals in the first period and 2,365 individuals in the second period.

To adjust for the disproportionate sampling of subgroups and for non-responses, we applied a weighting procedure for the estimation of mortality based on the GSOEP data. We follow the recommendation of Kroll and Lampert [30] that different weighting procedures should be used for those who are alive than for those who are dead. For individuals who are alive, the latest available cross-sectional weight is taken for all the times they were under observation. For deceased individuals, the weights are not available for the year of death, and the latest available weight proceeding the year of death is therefore taken.

We employ survival analysis and Gompertz regression models to estimate mortality rates, construct life tables, and estimate life expectancy at age 65. The Gompertz hazard at age *x* (*μ*_*x*_) can be expressed as follows:

$$\mu_{x}=ae^{bx},$$ (1)

where *a* denotes the level of mortality at the initial age and *b* denotes the rate of mortality growth over age.

The life table functions and the methods for constructing them are described in detail elsewhere [61].

The German Microcensus data are used to estimate the age-specific prevalence of long-term care for 2004 and 2012. The MC data are collected in an annual household survey that covers 1% of the population. As participation in the MC is obligatory by law, non-response is not an issue. In this paper, we use the 70% sample of the original MC dataset. These data cover individuals who were living not only in private households, but also in institutions. The receipt of LTC benefits for home care is considered as a proxy for needing care. While this is only one of several types of LTC benefits available, it accounts for the largest share. For instance, in 2015, 65% of all social LTC beneficiaries were receiving cash or non-cash benefits for home care [47]. In the 2004 MC, respondents were asked only whether they were receiving cash LTC benefits for self-organized nursing care assistance (so-called “Pflegegeld”). In the 2012 MC, the question on LTC benefits was slightly changed to ask respondents whether they were receiving not just monetary benefits (“Pflegegeld”), but non-cash LTC benefits for home care (“Pflegesachleistung”), or a combination of the two. More on the changes in this question can be found in the discussion part of the paper. Eligibility for benefits is determined based on a subjective physical assessment provided by the applicant, and on an evaluation of the applicant’s limitations in daily living activities. The LTC prevalence is defined as the proportion of people receiving benefits in a particular year.

Because the GSOEP data do not cover individuals living in institutions, the mortality rates were adjusted upward using the administrative data of the largest public health insurance fund, AOK. Based on a random sample of 250,000 people aged 50 or older, we estimated age-specific mortality rates for individuals living in institutions in 2010–2013. For the adjustments, we use the formula proposed by Vaupel and Jashin [62], in which the total mortality represents a sum of mortality for subgroups adjusted by the corresponding shares of these groups:

$$\bar{\mu }\left(x\right)=\pi \left(x\right)\mu_{1}\left(x\right)+\left[1-\pi \left(x\right)\right]\mu_{2}\left(x\right),$$ (2)

where $\bar{\mu }\left(x\right)$- observed hazard rates for the entire population; *μ*_1_(*x*) and *μ*_2_(*x*)- hazard rates for two sub-populations; *π*(*x*)- proportion of the population of one of the sub-groups in the total population.

Using the age-specific prevalence of needing long-term care and Sullivan’s method [63], we construct life tables and estimate long-term care-free life expectancy and life expectancy with LTC for three educational categories. In the computation of CFLE and CLE, we referred to the Practical Guide for the Health Expectancy Calculation prepared by the European Health and Life Expectancy Information System (EHLEIS) [64]. We referred to the Technical Report by Andreev and Shkolnikov [65] and applied the Monte Carlo simulations to approximate the standard errors and estimate the confidence limits for CFLE and CLE.

Life expectancy with long-term care needs is computed as the difference between the remaining life expectancy at age *x* and CFLE. The health ratio represents the proportion of care-free life expectancy in the total remaining life expectancy.

The level of educational attainment is used as an indicator of socioeconomic status. It is defined here as the highest educational degree the individual has earned. First, the highest educational degree obtained by each man is constructed and then collapsed into three categories based on the International Classification of Education (ISCED) [66]: *lower secondary education (“low”)*, *upper secondary education (“middle”)*, and *tertiary education (“high”)*. The cases for which absolutely no information is available are recoded as missing. In the GSOEP 1997–2004, about 9% of cases are of individuals who initially reported having a higher level of education, but later reported having a lower level of education. Such inconsistent cases are excluded from the analysis. Various categories were re-created to see whether a change in the categorization would change the results (not presented here), but the results remained largely consistent.

To evaluate the magnitude of the absolute and the relative educational inequalities in long-term care-free life expectancy, the Slope Index of Inequality (SII) [67] and the Relative Index of Inequality (RII) are computed [68]. In performing the computation, we referred to the handbook on health inequality monitoring prepared by the WHO [69]. Both indices are based on the regression and take the whole SES distribution into account, rather than comparing only the advantaged and the disadvantaged groups. To estimate these measures, the weighted sample of older men was first ranked by educational level from the lowest (rank 0) to the highest (rank 1) levels. The so-called rigid score was then estimated. This score represents a midpoint of the range in the cumulative distribution of the older individuals in the given educational category. Subsequently, the long-term care-free life expectancy for each educational category is regressed against the corresponding rigid score, and two predicted values of CFLE are calculated for the lowest and the highest ranks. As the distributions of educational categories differed slightly in the two surveys, the two distributions were pooled together for the estimation of the indices. The slope index of inequality is the absolute difference in the predicted value of CFLE between the top and the bottom educational ranking, while the relative index represents the rate ratio between two groups. A positive SII value reflects a higher prevalence of the health indicator in the most advantageous group. Similarly, when ranked from the least to the most educated (from rank zero to one), a RII value greater than one indicates a higher prevalence among the highly educated.

## Results

### Age-specific mortality

Table 1 summarizes the estimates of the age-specific mortality rates from the GSOEP data before and after the adjustments. After fitting the Gompertz model and taking the mortality of the institutionalized men into consideration, the results become reasonably close to the age-specific mortality rates estimated from the Human Mortality Database (HMD) data [70].

**Table 1:** Estimated and adjusted age-specific mortality rates; men aged 65 or older; Germany, 1997–2012.

	Empirical mortality rates from GSOEP		Estimated with Gompertz, GSOEP		Mortality rates from AOK, 2010–2013	Percent of people staying in institu-tions	Mortality rates adjusted on the AOK data		Mortality rates estimates from the HMD	
	1997–2004	2005–2012	1997–2004	2005–2012			1997–2004	2005–2012	1997–2004	2005–2012
65–69	0.026	0.017	0.022	0.015	0.163	1.37	0.024	0.017	0.024	0.019
70–74	0.037	0.022	0.037	0.026	0.244	1.43	0.040	0.029	0.038	0.025
75–79	0.054	0.041	0.063	0.044	0.315	2.41	0.069	0.051	0.061	0.043
80–84	0.068	0.081	0.106	0.075	0.361	4.30	0.117	0.087	0.101	0.073
85–89	0.207	0.090	0.176	0.126	0.442	7.98	0.198	0.152	0.172	0.124
90+	0.210	0.150	0.250	0.177	0.546	16.2	0.298	0.237	0.287	0.271

Source: Own estimates

According to the GSOEP estimates, the male age-specific mortality rates declined between the two periods when education is not considered.

Because of the lack of information in the health insurance data on the educational levels of individuals, we are unable to estimate the mortality rates for each educational category. Therefore, the age-specific mortality rates and the proportions of people living in institutions for each age group estimated from the AOK data are assumed to be the same for all educational categories.

When education is considered, the rates change differently for older men with different educational backgrounds (Table 2). Among the less educated men, the empirical rates declined for the 65–74 age group only; while among the highly educated men, mortality increased for the 75–84 age group only, and declined for all other age groups. As soon as the Gompertz model is employed, the results suggest that there was a decline in mortality levels between the two analyzed periods for all educational categories and age groups.

**Table 2:** Estimated and adjusted age-specific mortality rates by educational groups; men, aged 65 or older; Germany, 1997–2012.

	Empirical mortality rates from GSOEP		Estimated with Gompertz, GSOEP	
	1997–2004	2005–2012	1997–2004	2005–2012
**Low**				
65–69	0.043	0.028	0.026	0.023
70–74	0.044	0.017	0.044	0.038
75–79	0.064	0.074	0.072	0.065
80–84	0.049	0.083	0.120	0.108
85–89	0.180	0.181	0.197	0.180
90+	0.184	0.354	0.278	0.255
**Middle**				
65–69	0.022	0.023	0.024	0.017
70–74	0.035	0.031	0.039	0.028
75–79	0.063	0.032	0.066	0.047
80–84	0.081	0.076	0.109	0.080
85–89	0.234	0.105	0.179	0.134
90+	0.213	0.128	0.252	0.187
**High**				
65–69	0.022	0.005	0.015	0.011
70–74	0.026	0.010	0.026	0.018
75–79	0.028	0.041	0.043	0.031
80–84	0.055	0.091	0.072	0.052
85–89	0.119	0.031	0.119	0.088
90+	0.108	0.093	0.164	0.122

Source: Own estimates

Our findings also clearly illustrate that there is a mortality-education gradient: i.e., the higher the educational attainment, the lower the mortality. For the 1997–2004 period, the hazard ratio (HR) of death for the older men with low education was 1.101 (p = 0.608) relative to the men with middle education (reference category), and was 0.651 (p = 0.013) for the men with high education relative to the men in the reference category. In the second period, the HR was 1.366 (p = 0.058) for the less educated men relative to the men with middle education; while the HR for the highly educated men was identical to that in the first period under study, but with a p-value of zero.

### Age-specific prevalence of LTC

Table 3 below provides the prevalence of LTC use, which we estimated from the MC and from official statistics on social long-term care provided by the Federal Health Monitoring System [71].

**Table 3:** Age-specific prevalence rates for LTC use based on different data sources; men, aged 65 or older; Germany, 2004 and 2012 (percent).

Age groups	MC data		Data from the Federal Health Monitoring System	
	Based on recipients of cash benefits	Based on recipients of cash and non-cash benefits	Based on recipients of cash and non-cash benefits	
	2004	2012	2003	2013
65–69	1.3	1.5	2.0	2.1
70–74	2.0	2.5	3.5	3.2
75–79	3.2	3.9	5.9	5.9
80–84	6.3	7.3	10.5	10.7
85–89	10.0	13.8	16.2	17.3
90+	22.3	25.4	25.3	25.8

Source: Own estimates. In estimating the prevalence rates from the official statistics, we used the population exposures from the Human Mortality Database (HMD). Because official data on LTC benefits by type are available only every second year beginning in 1999, the numbers given here are for 2003 and 2013.

The comparison of LTC prevalence reveals that the percentages computed from the MC are, in general, lower than those computed from the official aggregated statistics. The variation is more pronounced for the first period, which might reflect the slight difference in the formulation of the question on the use of LTC in 2004 and 2012 (monetary vs. monetary and in-kind benefits). In general, the age-specific LTC prevalence estimated from the MC data shows that LTC use increased between 2004 and 2012, regardless of educational level.

**Fig 1:**
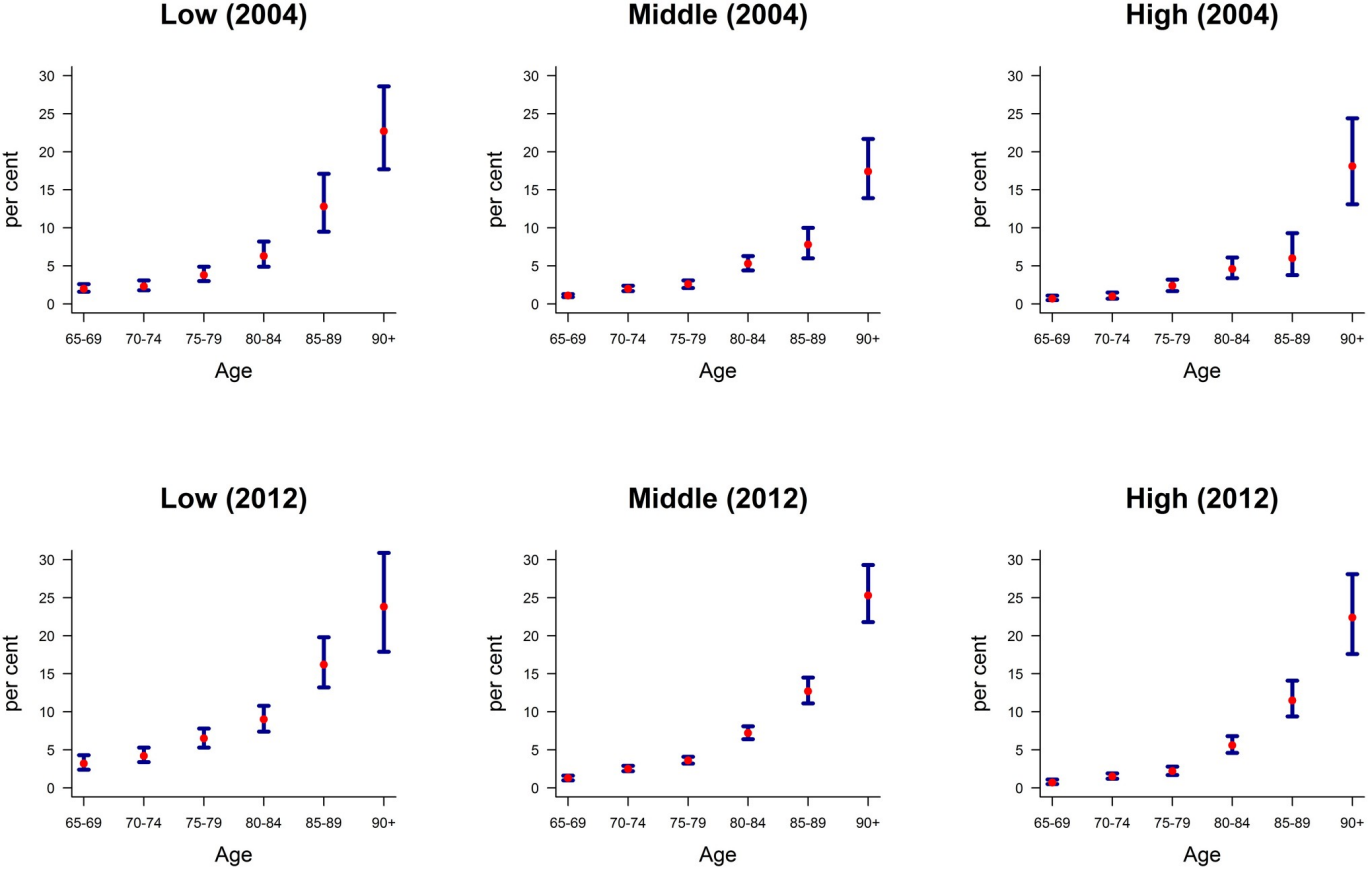
Age-specific prevalence (with 95% confidence intervals) of LTC use by educational group; men, aged 65 or older, Germany, 2004 and 2012 (percent). Source: Own estimates from the German Microcensus data, 2004 and 2012.

If we compare the LTC prevalence of the educational categories, we see that the prevalence is highest for men with low education, and declines with increasing educational level. The finding holds for both years (Fig 1).

### Trends in life expectancy

Life expectancy for men at age 65, which was estimated from the GSOEP data and was later adjusted for mortality among institutionalized men, was 15.12 years for the 1997–2004 period and was 17.32 years for the 2005–2012 period. These estimates fit reasonably well with those for the national population available in the HMD. For instance, LE at age 65 among men was 15 years in 1997, increased to 16.43 years in 2004, and then to 17.59 years in 2012.

A clear educational gradient in both LE and CFLE is observed among the older men. In 2004, life expectancy at age 65 among those with low education was 14.20 years, or 3.3 years lower than LE among highly educated men (Table 4). The respective CFLE for these two educational categories was 13.64 and 16.97 years. If we compare three educational groups, we see that the biggest differences in LE and CFLE were between the men with high education and with middle education (2.7 years for LE and 2.6 years for CFLE, respectively).

**Table 4:** LE, CFLE, and CLE (in years) and the health ratio by educational group; men aged 65 or older, 1997–2004 and 2005–2012.

	LE		CFLE		CLE		Health ratio
**1997–2004**	**value**	**Δ**	**value**	**Δ**	**value**	**Δ**	
Low	14.20		13.64		0.56		0.96
Middle	14.81	0.61	14.38	0.74	0.43	-0.13	0.97
High	17.49	2.68	16.97	2.59	0.52	0.09	0.97
High vs low		3.29		3.33		-0.03	
2005–2012	value	Δ	value	Δ	value	Δ	
Low	14.94		14.04		0.90		0.94
Middle	16.88	1.94	16.07	2.03	0.81	-0.09	0.95
High	19.54	2.66	18.63	2.56	0.91	0.11	0.95
High vs low		4.60		4.59		0.01	

Source: Own estimates. Δ represents the absolute difference between two neighboring educational categories; while high vs low Δ is the difference between the values for high and low education.

The differences between men with low and with middle education were about 0.6–0.7 years. When we look at life expectancy with LTC, we find very small differences between men with low and high educational attainment, but unexpectedly low values for men with middle education. The health ratio, which represents the share of care-free years in the remaining life expectancy, was only slightly lower for less educated than for highly educated men (0.96 vs 0.97).

**Fig 2:**
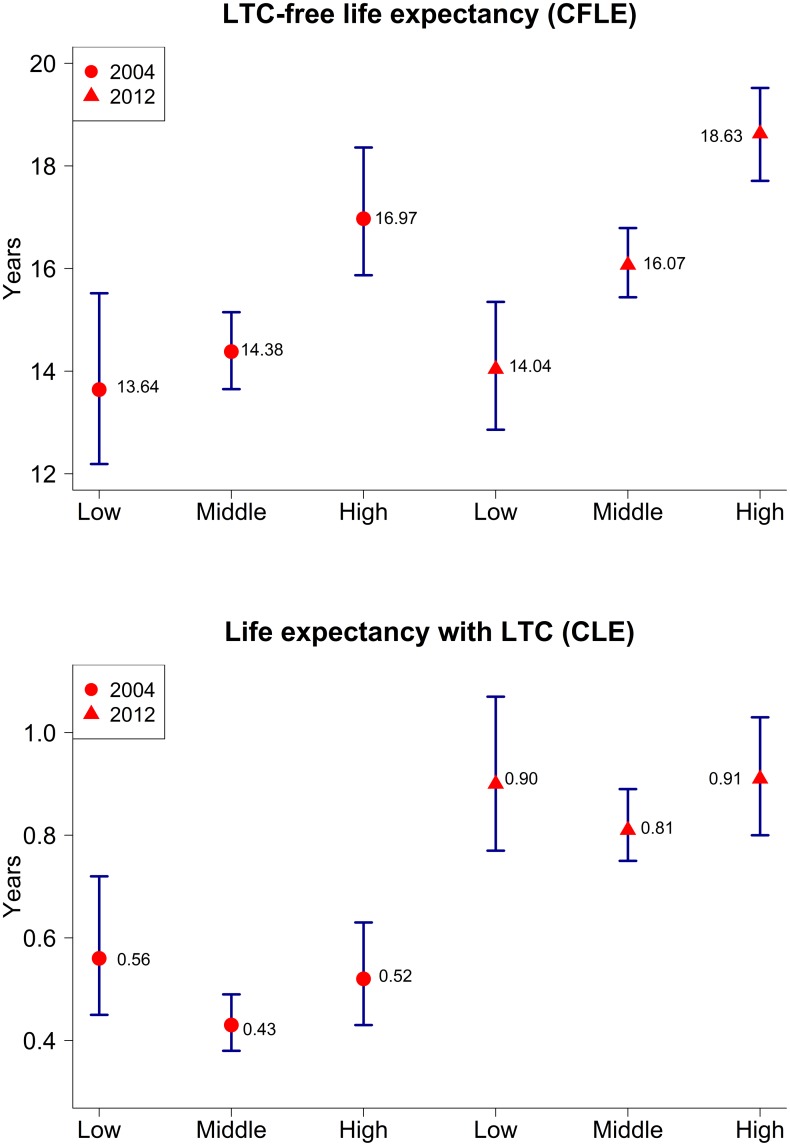
Long-term care-free life expectancy and life expectancy with care and 95% confidence intervals; Germany, men, aged 65 or older, 2004 and 2012. Source: Own estimates.

In 2012, a 65-year-old man with a high educational level could expect to live another 19.54 years, an estimated 18.63 years of which would be without the need for LTC. The educational gradient increased over time (Fig 2). By 2012, the difference in both LE and CFLE between men with high and low education had grown to about 4.6 years (14.94 vs. 19.54 in LE and 14.04 vs 18.63 in CFLE). However, the difference in LE and CFLE between men with high and middle education in 2012 was similar to that in 2004; i.e., about 2.6–2.7 years. Over the same period, the difference in LE and CFLE between men with low and middle education increased to around two years.

Between 2004 and 2012, CFLE grew by around 1.7 years among men with middle and high education, but by only 0.4 years among men with low education.

The number of years a 65-year-old man in 2012 could expect to live with LTC did not differ greatly depending on whether he had low or high education (around 0.9 years). But between 2004 and 2012, the number of years spent living with LTC increased slightly more among men with high than with low education. The health ratio declined over the study period for all educational categories.

Both the SII and the RII confirm that levels of education-based inequality in long-term care-free life expectancy among men increased between the two analyzed periods. For instance, in the first period under study, the CFLE of the most educated men was 1.3 times as high as that of the least educated; while in the second period under study, the ratio had increased to 1.4. In absolute term as well the SES gradient in CFLE increased between the two periods, from 4.28 years (p = 0.002) to 5.93 years (p = 0.000).

## Discussion

### Summary of findings

Based on three different individual-level datasets (GSOEP, Microcensus, and health insurance records), we assessed the relationship between educational attainment and long-term care-free life expectancy among older German men. We also investigated whether and, if so, how the education-LE/CFLE/CLE gradient changed between 2004 and 2012.

The available evidence suggests that there are substantial differences in mortality by socioeconomic status in Germany and elsewhere. To our knowledge, this is the first study that has provided estimates of long-term care-free life expectancy by educational level. Our results indicate that while disparities in LE and CFLE by educational status are increasing, when life expectancy with LTC is considered, the number of years with care needs has been increasing over time, but the variation across the educational groups has not been pronounced. This trend was found to be unchanged over time. Overall, the increase in the number of expected years of life without care and the decline in the health ratio of expected years of life without care to total expected years of life suggest that long-term care needs are expanding, regardless of the level of educational attainment.

Our estimates reveal that the education-LE and the education-CFLE gradients have been increasing over time: the difference in the number of years of CFLE between the lowest and highest educational groups increased by about 1.3 years (from about 3.3 years in 1997–2004 to almost 4.6 years in 2005–2012). The change in the education-CLE gradient was found to be insignificant.

The declining health ratio suggests, however, that while older German men are living longer, they are also becoming more likely to need LTC (expansion of LTC needs). This trend is occurring across educational levels. Among our more interesting findings is that the health ratio did not vary much between the educational groups. This means that there is no obvious and strong effect of education on the proportion of years with LTC needs, and that mortality does not differ much by educational group once people become dependent on care. It is the long-term care-free years of life that are influenced by SES.

Among the possible explanations for the diminishing educational gradient that can be observed as soon as older men start to need care is the postponement of morbidity (and disability) up to a certain age (e.g., 60 years) among men with higher SES. After these men grow older, their rates of health decline accelerate and their LTC needs increase more than is the case for men with lower SES. Thus, the health differences between men in different SES groups become smaller at older ages [72–73]. Another potential explanation for this pattern is selective mortality: i.e., that the most disadvantaged people die at earlier ages, while relatively robust individuals survive [72]. Moreover, social conditions might contribute to the decrease in SES inequalities at older ages, as the interplay of retirement and welfare state policies may slow down the decline in health among the most disadvantaged older adults [73].

Yet another possible explanation for the diminishing effect of education (or any other SES proxy) on LTC use is that when a person is in need of care, impending death becomes the largest contributing factor (and not socio-demographic or health factors). Gerstorf and colleagues [74], for instance, analyzed GSOEP data for people aged 70 years and above, and found that at higher ages, proximity to death is related to the loss of intellectual and sensory functioning, and that late-life changes in well-being are characterized by terminal decline. Given that people in need of care have limitations in physical and/or cognitive abilities, terminal decline might influence their use of LTC more than any of their socioeconomic characteristics.

In general, the results might be influenced by the indicators chosen for approximating SES (e.g., education, occupation, or income) and health (e.g., self-perceived health, cognitive impairment, disability, or long-term care need) [75]. The effect of income-related differences on individuals’ self-perceived health might produce different results than the impact of educational differences on the use of LTC.

Our findings are in agreement with those of Hoffmann and Nachtmann [76], who used official LTC statistics (micro data on recipients of benefits) for Germany. They found that between 1999 and 2005, there was an increase in the years spent in good health, but a decline in the share of healthy years relative to remaining life expectancy, which points to a relative expansion of morbidity. However, their analysis only looked at general trends in life expectancy, with no SES dimensions taken into account.

In another study on changes in LTC needs by small areas in Germany over the 2001–2009 period, Kreft and Doblhammer [44] split LTC into care levels. They found that there was an expansion of all care needs in the majority of the regions, but a compression of the most intensive care needs.

The expansion of morbidity hypothesis was supported in many countries by the findings of one of the earlier studies by Salomon and colleagues [77]. They used Global Burden of Disease data to estimate healthy life expectancy for 187 countries, and found that in most of these countries, an increase in overall life expectancy was followed by a rise in the number of healthy years lost to disability. In their discussion of the forces driving increasing healthy life expectancy, the authors highlighted reductions in child and adult mortality, rather than the decline in the number of years with disability.

When studying the SES-health association, it is important to determine whether the influence an individual’s SES has on health changes with age. The results of studies that have examined the development of the health-SES relationship over the life course, and particularly in later life, have been inconsistent. Schöllgen and colleagues [75] reviewed previous studies and reported that research has provided evidence for three contradictory hypotheses: the cumulative disadvantage theory (which posits that the effect of SES on health status increases with age), the age-as-leveler hypothesis (which argues that the impact of SES declines as the person becomes older), and the continuity theory (which posits that the SES-health relationship remains stable or changes little with age). The study that used data from the German Aging Survey has found that social inequalities in health in Germany have been either stable or increasing with age, depending on the SES and health indicators used.

Although our data do not allow us to conduct a cohort analysis, the issue of cohort trends in health should be given some attention. On the one hand, the cohorts in our study profited from the first educational expansion after World War I, and from having better career prospects after World War II [78]. On the other hand, they were born during periods characterized by recession and war, which have been shown to be associated with long-term negative consequences for health [79–81].

Recent research for Germany on the education-health association and, on how this association changes across individual lives and cohorts [82], has pointed to a rapid widening of the educational gap in health with age, and thus supports the cumulative advantage hypothesis. The results of this research also provide evidence of a divergence in health trajectories between educational groups in all of the analyzed cohorts, with the difference being more pronounced among the younger cohorts (referred to as the economic -wonder and baby -boom cohorts), and less pronounced among the older cohorts (referred to as pre-war, -war, -and–post-war cohorts). Another important finding is that the steeper health decline observed among men with lower education is mainly attributable to the cross-cohort trend, and that no cross-cohort differences can be found among highly educated men. This analysis was based on self-rated health among people aged 23–84.

Whether the same conclusions can be drawn when LTC needs are used as the indicator of an individual’s health is uncertain. As we mentioned above, the results of such an analysis can depend on the measures chosen for approximating health and SES. Further studies are needed to explore how the association between education and the need for long-term care changes when age and cohort are taken into account.

### Strengths and limitations

Compared to the previous studies conducted for Germany, this work has a number of advantages. First, it is based on two big datasets: the MC and the GSOEP. The Microcensus is an annual sample survey of German residents living in collective as well as in private households, which is not the case for many other surveys. The MC yields representative official statistics of the population, as participation is obligatory and the non-response rate is negligible. For our purposes, the most important advantage of using the MC is that it provides data on both long-term care use and educational attainment. LTC use is approximated by the receipt of long-term care cash and/or non-cash benefits for home care (“Pflegegeld” and “Pflegesachleistung”). This is a rather restricted and unbiased measure of an individual’s health status, since eligibility to receive these benefits is based on a physical assessment of the applicant (compared to, for instance, a self-perceived evaluation of health). It is also different from other approaches to disability measurement because it focuses on limitations in activities of daily living. The GSOEP dataset is smaller but longitudinal, and is the only source of information about mortality and educational levels among people aged 65 or older. Moreover, in the survey follow-ups, individual respondents are tracked even after they have moved to a new home.

Second, the variables related to the educational level are highly analogous in the GSOEP and the MC. In both datasets, these variables refer to the highest degree obtained, including general secondary, vocational, and higher education.

One of the limitations of the data in our analysis is that the GSOEP does not cover institutionalized population. We address this drawback by adjusting the mortality rates from the GSOEP data by the rates estimated for the institutionalized population from the health insurance data. However, this adjustment was based on the assumption that mortality among individuals living in institutions does not vary significantly across educational groups.

One of the shortcomings of the MC data is related to the change of the question on the use of long-term care services. As we mentioned above, the prevalence of LTC use was estimated based on responses to the question about the receipt of cash benefits (“Pflegegeld”). The German LTC insurance system offers the applicant a number of other long-term care benefits (e.g., in-kind benefits, day and night care, or a combination of cash and in-kind benefits). In 2004, participants were only asked whether they were receiving “Pflegegeld;” whereas in 2012, participants were asked whether they were receiving monetary and/or in-kind benefits. This discrepancy might lead to a slight underestimation of the prevalence of LTC use in 2004, which is difficult to evaluate. We estimate from official LTC statistics that throughout the analyzed period, monetary benefits accounted for about 80% of the combined cash and in-kind benefits. An earlier comparison of these benefits based on the official statistics [53] suggested that in 2004, other kinds of benefits or services may have already been reported as cash benefits [53]. In light of these findings, we can assume that the modification in the MC questionnaire should not cause very large differences in the estimation of CFLE and CLE, and that the results for 2004 and 2012 are therefore comparable.

Another constraint is related to the possible omission of some individuals in need of LTC in our analysis, since not everyone can fulfill the eligibility criteria as defined by the LTC insurance laws. Prior to 2013, the criteria were based on the functional limitations of an applicant, while individuals with cognitive problems were often neglected. But since our estimates based on the MC are in agreement with those of the official LTC statistics, any possible omissions should not influence our conclusions.

Finally, for several reasons, our analysis did not cover the female population. First, it is well-known that the educational differentials in morbidity and mortality are greater among men than among women [83–85]. For women, factors other than education can have a large impact on their care needs (e.g., marital status, household/family composition, place of residence, spouse’s SES or income, and behavioral factors), and these factors should be seen as confounders in the analysis. An earlier study for Germany found that education had an effect on long-term care among women [53], but only when the prevalence (not the incidence) of care was considered. Second, among the drawbacks of using education as the measure of SES is its skewness among the older age groups [84]. Given that the mortality rates estimated from the GSOEP for the oldest age groups were somewhat lower than for the national population, splitting deaths by educational groups might lead to erratic results, particularly for women with high levels of education. In our study, the estimates of LE for women looked rather inconsistent for the second period of analysis (see S1 Appendix). Unfortunately, there is no clear explanation for why this was the case, except that it might be purely a data problem. While we know that the educational structures of men and women differ, this gap was found to be persistent throughout the analyzed period, and not just between 2005 and 2012. Several educational categories were introduced to check whether the changes in the educational structure over time were affecting the estimates of mortality rates by SES, but this did not eliminate the problem. In general, we can state that the GSOEP data are not designed to estimate mortality. Despite the fact that information on a diseased individual is often obtained even if he or she moved before death, the number of deaths might be underestimated [30]–especially when mortality levels are estimated for groups with small numbers of observations, such as highly educated women.

## Conclusion

To develop effective health policies and strategies that ensure the fair allocation of limited resources among the growing number of older people with care needs, we must take the projected increase in the number of future LTC beneficiaries into account, and gain a better understanding of how the education-LE/CFLE/CLE gradient evolves over time. Further research is needed to address this issue among women. Additional research on cohort-related improvements in health and on changes in educational levels across cohorts would help to shed more lights on the situation in Germany.

## Supporting information

S1 Appendix Results for women. (PDF)
