# Supplementary material for: Changing educational gradient in long-term care-free life expectancy among German men, 1997-2012
Source: PLoS One. 2019 Sep 19;14(9):e0222842. doi: 10.1371/journal.pone.0222842 (PMC6752958; doi:10.1371/journal.pone.0222842)
Supplement: S1 Appendix — (PDF) [file pone.0222842.s001.pdf]

Table 1. Estimated and adjusted age-specific mortality rates; women 65 years and older; Germany, 1997-2012

	Empirical mortality rates from GSOEP		Estimated with Gompertz, GSOEP		Mortality rates from AOK, 2010-2013	Percent of people staying in institutions	Mortality rates adjusted on the AOK data		Mortality rates estimates from the HMD	
	1997-2004	2005-2012	1997-2004	2005-2012			1997-2004	2005-2012	1997-2004	2005-2012
65-69	0.013	0.006	0.012	0.007	0.135	0.91	0.013	0.008	0.011	0.009
70-74	0.024	0.015	0.022	0.014	0.167	1.50	0.024	0.016	0.020	0.016
75-79	0.043	0.026	0.041	0.027	0.208	3.35	0.046	0.033	0.036	0.030
80-84	0.063	0.051	0.075	0.054	0.242	7.50	0.088	0.068	0.068	0.059
85-89	0.092	0.076	0.139	0.105	0.279	15.00	0.160	0.131	0.127	0.113
90+	0.242	0.166	0.207	0.160	0.379	30.97	0.260	0.228	0.239	0.239

Source: Own estimates

Table 2. Estimated and adjusted age-specific mortality rates by educational groups; women, 65 years and older; Germany, 1997-2012

	Empirical mortality rates from GSOEP		Estimated with Gompertz, GSOEP	
	1997-2004	2005-2012	1997-2004	2005-2012
Low<br>65-69	0.010	0.006	0.014	0.007
70-74	0.022	0.016	0.026	0.013
75-79	0.049	0.030	0.048	0.026
80-84	0.095	0.038	0.088	0.052
85-89	0.094	0.091	0.159	0.102
90+	0.338	0.141	0.237	0.156
Middle<br>65-69	0.014	0.005	0.010	0.007
70-74	0.025	0.015	0.019	0.014
75-79	0.041	0.024	0.036	0.029
80-84	0.033	0.065	0.065	0.057
85-89	0.098	0.069	0.119	0.112
90+	0.153	0.192	0.174	0.171
High<br>65-69	0.015	0.006	0.009	0.006
70-74	0.023	0.017	0.016	0.012
75-79	0.023	0.026	0.029	0.025
80-84	0.055	0.041	0.054	0.049
85-89	0.027	0.043	0.098	0.096
90+	0.107	0.221	0.143	0.146

Source: Own estimates

Hazard ratio: middle education – reference category

1997-2004 low education: 1.358 (0.029); high education: 0.822 (0.466)

2005-2012 low education: 0.910 (0.508); high education: 0.853 (0.488)

Table 3. Age-specific prevalence rates in using LTC based on different data sources; women, 65 years and older; Germany, 2004 and 2012 (percent)

Age groups	MC data		Data from the Federal Health Monitoring System	
	Based on recipients of cash benefits	Based on recipients of cash and non-cash benefits	Based on recipients of cash and non-cash benefits	
	2004	2012	2003	2013
65-69	1.4	1.4	1.6	1.8
70-74	2.3	2.5	3.0	2.9
75-79	4.1	4.6	5.6	6.0
80-84	9.2	10.3	11.1	12.0
85-89	17.0	20.6	17.5	19.4
90+	34.0	38.4	26.0	25.6

Source: Own estimates; In the estimation of the prevalence rates from the official statistics, the population exposure was used from the Human Mortality Database (HMD); Official data on the benefits of LTC by types are available only every second year beginning from 1999 and thus the numbers here are given for the 2003 and 2013.

Table 4. LE, CFLE, and CLE (in years) and health ratio by educational groups; women 65 years and above, 1997-2004 and 2005-2012

1997-2004	LE		CFLE		CLE		Health ratio
	value	Δ	value	Δ	value	Δ	
Low	17.00	-	16.19	-	0.82	-	0.95
Middle	18.76	1.76	17.88	1.69	0.88	0.06	0.95
High	19.85	1.09	18.83	0.95	1.02	0.14	0.95
High vs low		2.85		2.64		0.20	
2005-2012							
Low	20.28	-	18.55	-	1.73	-	0.91
Middle	19.79	-0.49	18.54	-0.01	1.25	-0.48	0.94
High	20.60	0.81	19.35	0.81	1.26	0.01	0.94
High vs low		0.32		0.80		-0.47	

Source: Own estimates; Δ represents the absolute difference between two neighboring educational categories; high vs low Δ is the difference between values for high and low education

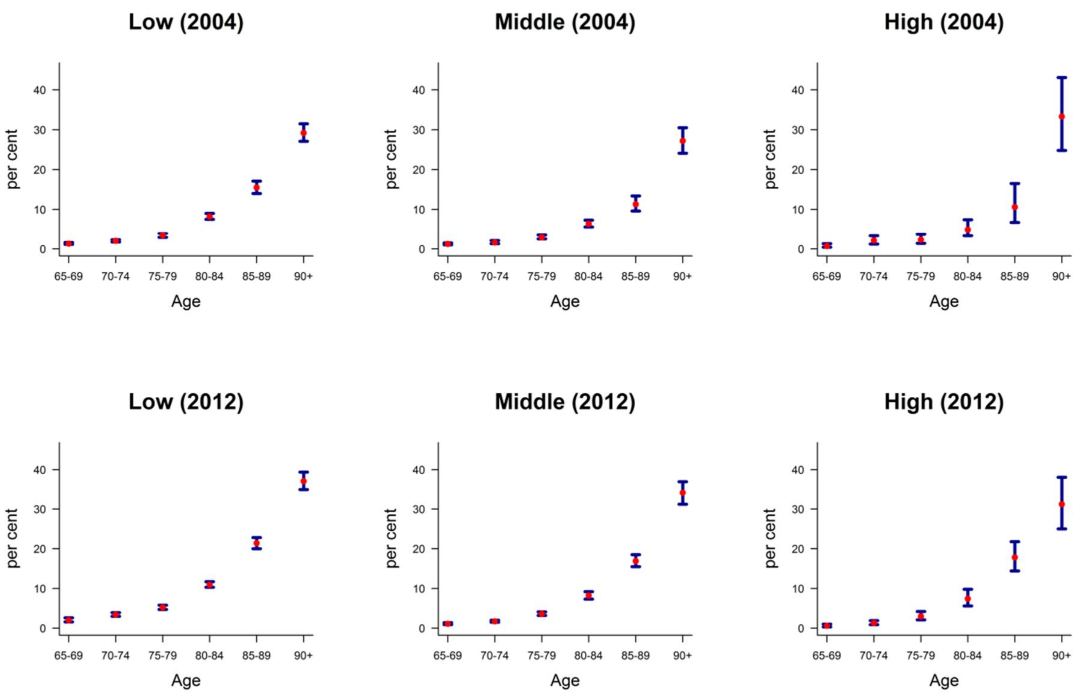

Fig 1. Age-specific prevalence (with 95% CI's) of using LTC by educational groups; women, 65 years and older, Germany, 2004 and 2012 (percent)

Source: Own estimates from the German Microcensus data, 2004 and 2012

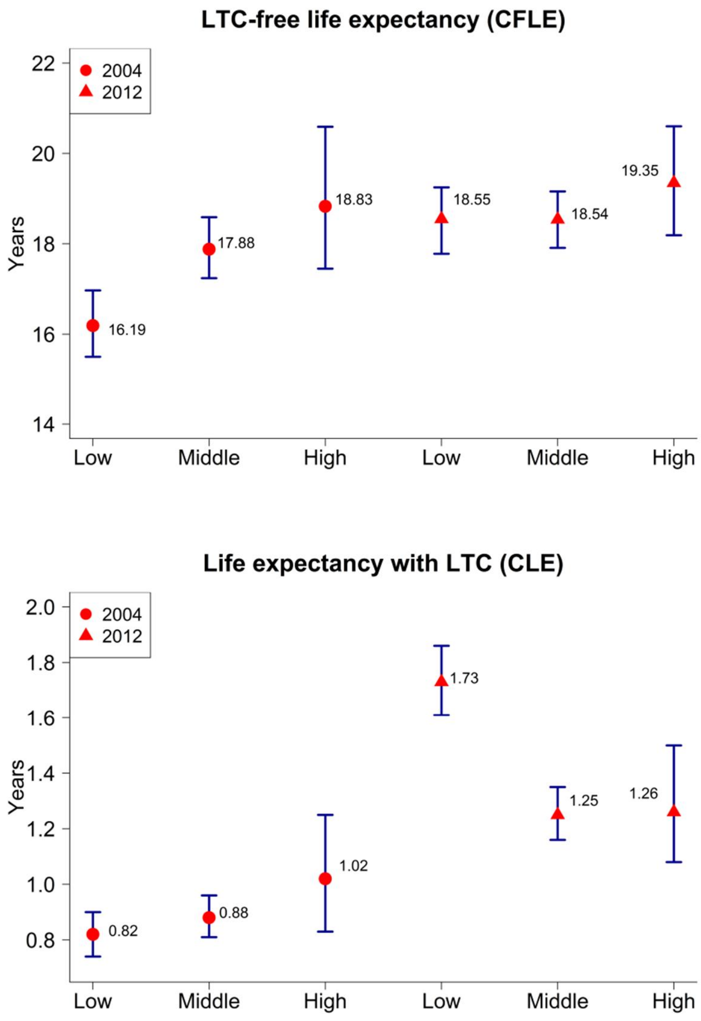

Fig 2. Long-term care-free life expectancy (CFLE) and life expectancy with care (CLE) and 95% confidence intervals; Germany, women, aged 65 or older, 2004 and 2012
